# The Seroprevalence of Hepatitis C Antibodies in Immigrants and Refugees from Intermediate and High Endemic Countries: A Systematic Review and Meta-Analysis

**DOI:** 10.1371/journal.pone.0141715

**Published:** 2015-11-11

**Authors:** Christina Greenaway, Ann Thu Ma, Lorie A. Kloda, Marina Klein, Sonya Cnossen, Guido Schwarzer, Ian Shrier

**Affiliations:** 1 Division of Infectious Diseases, Jewish General Hospital, McGill University, Montreal, Canada; 2 Centre for Clinical Epidemiology, Lady Davis Research Institute for Medical Research, Jewish General Hospital, Montreal, Canada; 3 Department of Epidemiology and Biostatistics, McGill University, Montreal, Canada; 4 Department of Internal Medicine, Centre Hospitalier de l’Université de Montréal, Montreal, Canada; 5 Library, McGill University, Montreal, Canada; 6 Division of Infectious Diseases, McGill University Health Center, McGill University, Montreal, Canada; 7 Institute of Medical Biometry and Statistics, Medical Centre - University of Freiburg, Freiburg, Germany; University of New South Wales, AUSTRALIA

## Abstract

**Background & Aims:**

Hepatitis C virus (HCV) infection is a significant global health issue that leads to 350,000 preventable deaths annually due to associated cirrhosis and hepatocellular carcinoma (HCC). Immigrants and refugees (migrants) originating from intermediate/high HCV endemic countries are likely at increased risk for HCV infection due to HCV exposure in their countries of origin. The aim of this study was to estimate the HCV seroprevalence of the migrant population living in low HCV prevalence countries.

**Methods:**

Four electronic databases were searched from database inception until June 17, 2014 for studies reporting the prevalence of HCV antibodies among migrants. Seroprevalence estimates were pooled with a random-effect model and were stratified by age group, region of origin and migration status and a meta-regression was modeled to explore heterogeneity.

**Results:**

Data from 50 studies representing 38,635 migrants from all world regions were included. The overall anti-HCV prevalence (representing previous and current infections) was 1.9% (95% CI, 1.4–2.7%, I^2^ 96.1). Older age and region of origin, particularly Sub-Saharan Africa, Asia, and Eastern Europe were the strongest predictors of HCV seroprevalence. The estimated HCV seroprevalence of migrants from these regions was >2% and is higher than that reported for most host populations.

**Conclusion:**

Adult migrants originating from Asia, Sub-Saharan Africa and Eastern Europe are at increased risk for HCV and may benefit from targeted HCV screening.

## Introduction

Hepatitis C virus (HCV) infection is a serious global health threat with an estimated 150–170 million individuals chronically infected worldwide, resulting in 350,000 deaths each year due to associated cirrhosis and hepatocellular carcinoma (HCC) [1–3]. Mortality due to HCC has increased over the past four decades in many countries and in part is due to chronic HCV. [4, 5] Chronic HCV has also resulted in an enormous economic burden and lost productivity.[6] HCV infected individuals often remain asymptomatic for 30 years or more until liver disease is advanced [7]. Early detection therefore is critical as treatment usually leads to viral eradication, prevents progression of liver disease, and decreases all-cause mortality [8]. The recent development of safer, more tolerable and highly effective direct acting antiviral combinations offers the real possibility of cure for all HCV infected patients [8, 9]. This provides a clear and compelling rationale for identifying and screening groups at risk to avert the projected individual and economic burden from HCV.

The traditional approach to HCV control in most low prevalence countries is to screen groups with behavioral risk factors for exposure to infected blood, such as through intravenous drug use or receipt of blood products prior to routine screening. In spite of these programs, the majority of individuals with HCV (45–80%) in these countries remain undiagnosed and unaware of their infection until they develop chronic liver disease [10, 11]. To address this issue in the US, the Centre for Disease Control and Prevention (CDC) and the U.S. Preventive Services Task Force (USPTF) recently recommended a one-time HCV birth cohort screening program (Baby Boomers born between 1945 and 1965) in addition to risk factor based screening programs [10, 12].

Migrants born in intermediate and high HCV prevalence countries who live in low HCV prevalence countries are likely to be at increased risk for HCV due to exposure in their countries of origin [13]. Unlike low HCV prevalence countries where the primary mode of transmission is through intravenous drug use, most infections in intermediate and high HCV endemic countries are acquired iatrogenically through contaminated needles, medical procedures or receipt of unscreened contaminated blood products [7, 14]. Most migrants are therefore unlikely to be detected in current HCV screening programs. Furthermore they have not been identified as a group that should be targeted for HCV screening with the exception of recent UK and Canadian guidelines.[13, 15] This is primarily due to the fact that the HCV burden in this population has not been adequately quantified.

To address this knowledge gap, we carried out a systematic review and meta-analysis on the seroprevalence of HCV in migrants living in several different low HCV prevalence, high migrant-receiving host countries.

## Materials and Methods

### Data sources and searches

This article was prepared and reported according to PRISMA guidelines (S1 Appendix) [16]. Four electronic databases, including Medline, Medline In-Process, EMBASE, and the Cochrane Database of Systematic Reviews were searched from inception until June 17, 2014. The search strategy was developed by a medical librarian and the strategy and search terms for MEDLINE are listed in supporting information (S2 Appendix). In summary, search terms included those for hepatitis C and the population of interest (migrants, foreign born, immigrants, refugees, asylum seekers) using a combination of text words and subject headings appropriate to each database. No limits by date or language were applied to the search. Additional studies were identified by examining the bibliographies of eligible studies and review articles.

### Study selection and quality assessment

Original studies that reported data on the anti-HCV prevalence in migrants originating from low/intermediate income, intermediate/high HCV prevalence countries, and arriving in high income, low/intermediate HCV prevalence countries, were included. Conference abstracts and proceedings were not included due to concerns regarding the inability to determine the quality of the methods. We adapted the GRADE method to assess the quality of the body of evidence and assessed the risk of bias, inconsistency, indirectness, and imprecision of the data [17, 18] (S3 Appendix). We did not assess small study effects (e.g. publication bias) as this is not reliable for seroprevalence studies [19].

We only included studies judged to have a low to moderate risk of selection or detection bias. Studies were included if the entire population or a random sample of individuals captured in a particular settings such as clinics conducting immigrant and refugee screening, general primary care or medical clinics, and prenatal clinics over a set period of time and all participants were offered HCV blood testing. Studies were excluded if; 1) If a non-random sample of individuals from a site were recruited or if only selected individuals had HCV serologic testing performed, 2) surveys in which <65% of eligible persons agreed to participate in the study, and 3) the study population focused on migrants populations judged to be at either at lower risk (for example healthy blood donors [20–22]) or increased risk for HCV due to specific lifestyles or environmental conditions (i.e. sex workers, intravenous drug use, incarcerated migrants, men having sex with men (MSM), individuals being treated for chronic liver disease).

### Data extraction

Two authors screened the titles and abstracts of all identified articles, reviewed and selected full-text articles, and independently extracted and entered data from each included study into a database. Data on study design, recruitment method, serologic screening test used, confirmatory test used, study duration, country of landing, mean or median age, gender, migrants’ region of origin, and migration status were extracted.

The primary study outcome was the proportion of subjects with the presence of HCV antibody (anti-HCV) detected by an enzyme immunoassay (EIA) with or without reported confirmatory testing (previous and current infection). Categories of HCV antibody seroprevalence for individual countries were defined as: very low (<1%), low (1- <2%), intermediate (2-<3%), high (3-<5%), and very high (≥ 5%). Age was defined as the mean or median age of the study population categorized into the following groups: ≤18 years, 19–29 years, and ≥ 30 years. Studies on children were classified as those where all study subjects were ≤ 18 years of age. Region of origin and income level were defined according to the World Bank classifications [23]. Regions of origin were categorized as: Latin America and the Caribbean, Eastern Europe and Central Asia, Middle East and North Africa, Sub-Saharan Africa, South Asia, and East Asia and the Pacific Categories for ‘Combined Africa’ and ‘Combined Asia’ were added to the region of origin variable, so that studies that only classified migrants as originating from Africa or Asia could be included. Studies with participants from several regions of origin were described as “Mixed”, however anti-HCV prevalence by region of origin was estimated if the data were reported this manner. Migration status was classified as; ‘immigrants’ (immigrants and adopted children), ‘refugees’ (refugees and asylum seekers), and ‘other’ (mixed populations or migrant status not mentioned).

### Data synthesis and analysis

For each study, the prevalence of HCV antibodies (includes previous and current infection) and viremic HCV infections were calculated as the reported numbers of subjects positive for anti-HCV or HCV PCR, respectively, divided by the total number of subjects screened for each of these markers. Proportions were transformed with the logit transformation and pooled using a random-effects model [24, 25]. The logit transformation was used to avoid studies with few events from being weighed too heavily in the random-effects model and the multivariate analysis, which used a random-effects logistic regression model, is also based on the logit transformation [26]. The logit transformation, arcsine and Freeman-Turkey methods, different methods used in meta-analysis of proportions were compared in a sensitivity analysis. Overall heterogeneity among studies was assessed using the *I* ^2^ statistic and estimates were stratified by age group, region of origin and migration status, as these variables were believed *a priori* to be important predictors of chronic HCV infection [27]. The meta-analysis was performed using the *metaprop* command of the meta package (3.6–0) in R (version 3.1.3) [28]. A random-effects meta-regression, using the *glmer* command of the lme4 package (version 1.0–4) to fit generalized linear mixed-models in R, was used to further explore heterogeneity. Unadjusted models for each of age group (reference: ≤ 18 years), migrant status (reference: immigrants), and region of origin (reference: Latin America & Caribbean) with anti-HCV prevalence as the outcome were run (see Table 1 for description of each categorical variable). Two adjusted meta-regression models were run and included; 1) age and migrant status, and 2) migrant status and region of origin. It was not possible to create a model to adjust for age by region of origin because the age of migrants was not stratified by region of origin in the included studies [29]. We created maps of country specific anti-HCV prevalence with data from the WHO [30] or more recent estimates [31–35] using the ArcGIS-9 software (ESRI data and Maps 9·3, 2008; USA). Weighted regional HCV seroprevalence (categorized by World Bank classification) were calculated from these same country specific seroprevalence data and multiplied by the 2009 country specific population estimates from the World Bank (Tables A and B in S4 Appendix) [23]. Regional seroprevalence estimates from our meta-analysis were superimposed on the same map for comparison.

**Table 1:** Characteristics of included studies [70–119]. Abbreviations: EIA, enzyme immunoassay; CIA, chemiluminescent immunoassay; PCR, polymerase chain reaction.

	Number of studies (%)	Sample size (%)
**Total**	50 (100)	38 635 (100)
**Age group** ^a^		
Reported	**35 (70.0)**	19 309 (50.0)
≤ 18 years	11 (31.4)	3767 (19.5)
19–29 years	15 (42.9)	9172 (47.5)
≥ 30 years	9 (25.7)	6370 (33.0)
Not reported	15 (30.0)	19 326 (50.0)
**Population Characteristics**		
Children^b^	9 (18.0)	2452 (6.3)
Pregnant Women	8 (16.0)	8347 (21.6)
General Population	33 (66.0)	27 838 (72.1)
**Migration status**		
***Immigrant***	29 (58.0)	17 800 (46.1)
Immigrant	21 (42.0)	16 275 (42.1)
Adopted child	7 (14.0)	1350 (3.4)
Foreign student	1 (2.0)	175 (0.5)
***Refugee***	11 (22.0)	5186(12.3)
Refugee	10 (20.0)	4915 (13.4)
Asylum seeker	1 (2.0)	271 (0.7)
***Other***	10 (20)	15649 (40.5)
Mixed immigrant and refugee population	6 (12.0)	11 350 (29.4)
Not specified	4 (8.0)	4299 (11.1)
**Region of origin** ^c^		
Latin America and Caribbean	13 (12.5)	5240 (13.6)
Eastern Europe and Central Asia	25 (24.0)	7505 (19.4)
Middle East and North Africa	11 (10.6)	2681 (6.9)
Sub-Saharan Africa	15 (14.4)	4446 (11.5)
South Asia	4 (3.8)	354 (0.9)
East Asia and Pacific	7 (6.7)	1007 (2.6)
Combined Africa	9 (8.7)	2095 (5.4)
Combined Asia	10 (9.6)	1107 (2.9)
Mixed	10 (9.6)	14 200 (36.8)
Total^d^	104 (100)	38 635 (100)
**Country of landing**		
Australia	3 (6.0)	7939 (20.5)
Canada	2 (4.0)	339 (0.9)
France	3 (6.0)	1911 (4.9)
Germany	1 (2.0)	1025 (2.7)
Greece	6 (12.0)	5146 (13.3)
Israel	3 (6.0)	1480 (3.8)
Italy	11 (22.0)	5011 (13.0)
Netherlands	2 (4.0)	1239 (3.2)
Spain	15 (30.0)	11 915 (30.8)
Sweden	1 (2.0)	88 (0.2)
United States	3 (6.0)	2542 (6.6)
**Study Setting for Screening**		
Adopted Children	7 (14.0)	1350 (3.5)
Migrant screening^e^	15 (30.0)	14 060 (36.4)
Prenatal screening	9 (18.0)	9328 (24.1)
General clinics	12 (24.0)	6832 (17.7)
Tropical medicine	4 (8.0)	3827 (9.9)
Surveys	3 (6.0)	3238 (8.4)
**Decade of study publication**		
1990–1999	14 (28.0)	7655 (19·8)
2000–2009	27 (54.0)	22518 (58·2)
2010–2013	9 (18.0)	8462 (22·0)
**Diagnostic method**		
EIA/CIA alone	28 (56.0)	20820 (53.9)
EIA/CIA + confirmatory method	22 (44.0)	17814 (46.1)
**EIA generation**		
1^st^ generation	1 (2.0)	88 (0.2)
2^nd^ generation	12 (24.0)	6632 (17.2)
3^rd^ generation	37 (74.0)	31915 (82.6)
**PCR**	6^f^	5139 (13.3)

^a^ Age group based on mean or median age. Only 35 of the 50 studies included data on age. The remaining studies reported no information on age specific to the migrant participants or included a range that overlapped more than one age group.

^b^ Studies where all migrants were ≤ 18 years were considered to be studies of children.

^c^ According to World Bank classification

^d^ The number of studies exceeds the 50 studies included in this review and meta-analysis because a single study could report seroprevalence in multiple regions of origin.

^e^ Migrants detained upon arrival by boat.

^f^ Of the nine studies (N = 5932), only six performed PCR on all anti-HCV positive participants (N = 5139)

## Results

**Fig 1:**
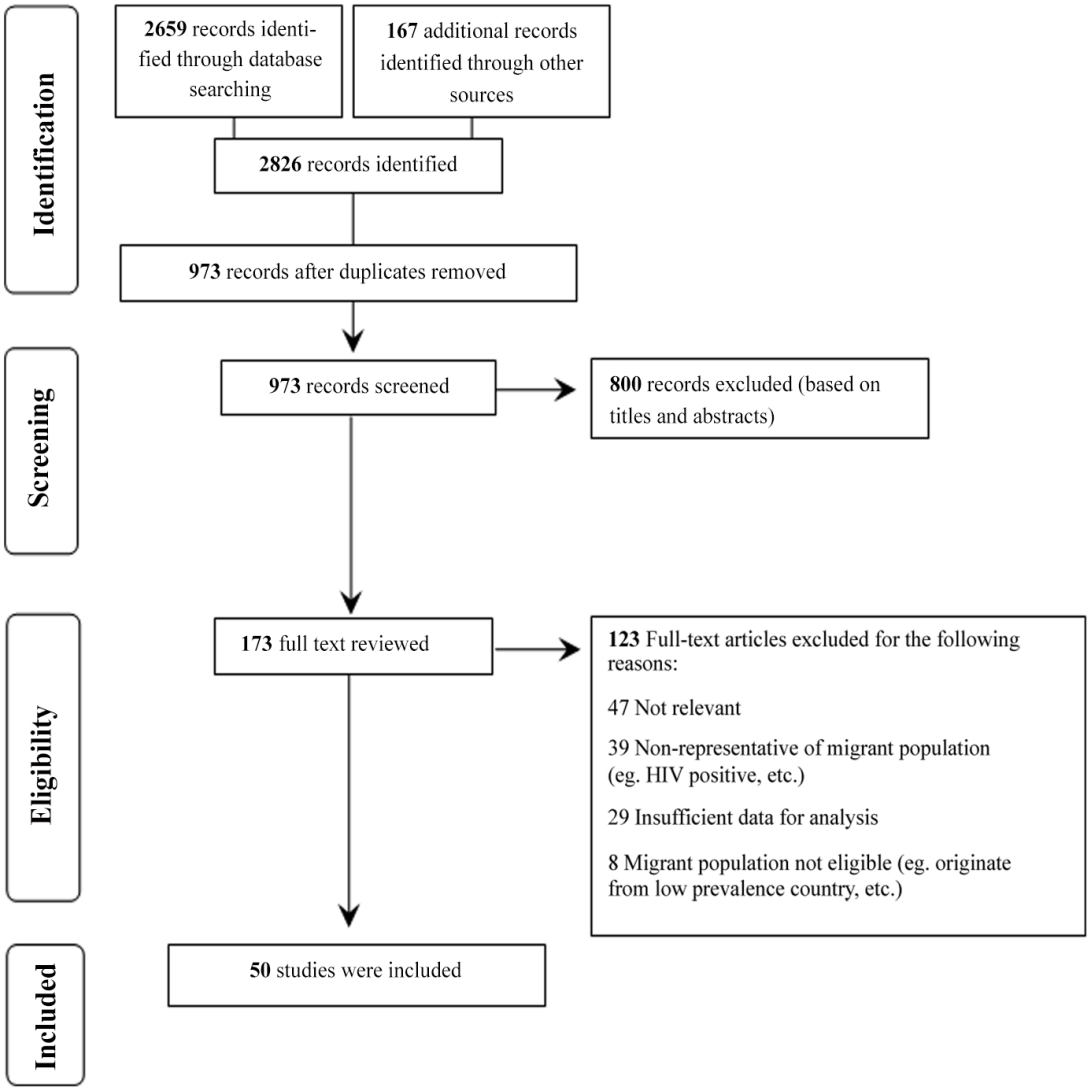
Flowchart of the identification, screening, and inclusion/exclusion of studies.

A total of 973 titles and abstracts were screened, 173 full text articles were reviewed, and 50 were included in this study (Fig 1). Included studies reported on 38 635 participants from all world regions who arrived in Australia, Canada, Europe, Israel and the United States (Table 1). Across all studies, there was a low to moderate risk of bias, moderate to high inconsistency, moderate imprecision, and low indirectness. We therefore considered the overall quality of the body of data to be low to moderate (S3 Appendix). Although all studies measured HCV antibodies, only 44% (N = 22) of studies reported confirmatory testing. The prevalence of antibodies, was similar in those studies in which a confirmatory test was reported or not [2.1% (95% CI, 1.2–3.6) vs. 1.8% (95% CI, 1.1–2.8%), respectively] (data not shown).

All studies were therefore included in the meta-analysis, and resulted in an overall anti-HCV prevalence of 1.9% (95% CI, 1.3–2.7) (Table 2). A sensitivity analysis was performed and compared the overall anti-HCV prevalence estimates using the logit transformation, arcsine and Freeman-Turkey and all yielded similar results [1.91 (95% CI, 1.35–2.69), 2.2 (95% CI, 1.4–3.1) and 2.1 (95% CI, 1.4–3.0) respectively] [26]. Only six studies (N = 5139 individuals) reported results of HCV PCR testing. In these studies, 75% of individuals were found to have viremic infection (i.e. HCV RNA was detected). Significant heterogeneity was found in the overall pooled HCV seroprevalence estimate (I^2^, 96.1% [95% CI, 95.5–96.7]) that decreased but was still at least moderate when data were stratified by age and region of origin, known important predictors of HCV seroprevalence (Table 2).

**Table 2:** Pooled HCV seroprevalence overall and stratified by migration status, region of origin, and age. Abbreviations: CI, confidence interval.

	N studies	N subjects	Seroprevalence % (95% CI)	I^2^% (95% CI)
**Overall**	50	38 635	1.9 (1.4, 2.7)	96.1 (95.5, 96.7)
**Overall (without children)**	41	36 183	2.2 (1.6, 3.2)	96.7 (96.1, 97.2)
**Age** ^a^				
≤ 18 years	11	3767	0.8 (0.4, 1.6)	63.8 (30.8, 81.1)
19–29 years	15	9172	2.0 (1.3, 3.1)	88.5 (82.7, 92.4)
≥ 30 years	9	6370	5.6 (3.7, 8.5)	93.6 (89.9, 95.9)
**Population category**				
Children^b^	9	2452	0.6 (0.3, 1.3)	16.1 (0, 58.2)
Pregnant Women	8	8347	2.0 (1.3, 3.1)	85.5 (73.3, 92.1)
General Population	33	27838	2.3 (1.6, 3.5)	96.8 (96.2, 97.3)
**Migrant status**				
Immigrants	29	17 800	2.3 (1.5, 3.5)	95.9 (94.9, 96.7)
Refugees	11	5186	1.3 (0.8, 2.2)	69.4 (42.9, 83.6)
Other^c^	10	15 649	2.2 (1.2, 3.9)	95.8 (93.9, 97.1)
**Region of origin**				
Latin America & Caribbean	13	5240	0.7 (0.5, 1.1)	19.6 (0, 57.6)
Eastern Europe & Central Asia	25	7505	2.2 (1.3, 3.8)	89.3 (85.5, 92.1)
Middle East & North Africa	11	2681	1.4 (0.8, 2.4)	38.3 (0, 69.6)
Sub-Saharan Africa	15	4446	4.4 (2.6, 7.4)	91.5 (87.7, 94.2)
South Asia	4	354	4.8 (0.9, 21.3)	69.7 (12.7, 89.5)
East Asia & Pacific	7	1007	1.6 (0.9, 2.7)	0 (0, 53.5)
Combined Africa	9	2095	4.6 (2.7, 7.7)	79.2 (61.1, 88.9)
Combined Asia	10	1107	3.8 (2.3, 6.3)	39.4 (0, 71.1)
**Setting for screening**				
Adopted kids	7	1350	0.7 (0.4, 1.5)	0 (0, 0.6)
Migrant screening	15	14 060	1.3 (0.9, 2.0)	80.5 (68.7, 87.8)
General clinics	28	23 225	2.9 (1.9, 4.2)	96.7 (96.0, 97.3)
**Decade of publication**				
1990–1999	14	7655	2.8 (1.6, 4.7)	93.2 (90.3, 95.3)
2000–2009	27	22518	1.6 (1.0, 2.3)	90.9 (88.0, 93.1)
2010–2013	9	8462	2.4 (1.3, 4.6)	96.8 (95.4, 97.8)
**EIA test generation**				
1^st^ generation	1	88	2.3 (0.6, 8.6)	-
2^nd^ generation	12	6632	2.2 (1.4, 3.5)	86.9 (78.9, 91.8)
3^rd^ generation	37	31915	1.8 (1.2, 2.8)	96.8 (96.2, 97.3)

^a^ Only 35 of the 50 studies provided information regarding age of the study population and age groups were classified by mean or median age of the study population.

^b^ Population Category included all studies: Children (all subjects were <18 years), pregnant women, general population (mixed ages including some children).

^c^ The ‘Other’ subgroup includes studies where results were not stratified by migrant status.

**Fig 2:**
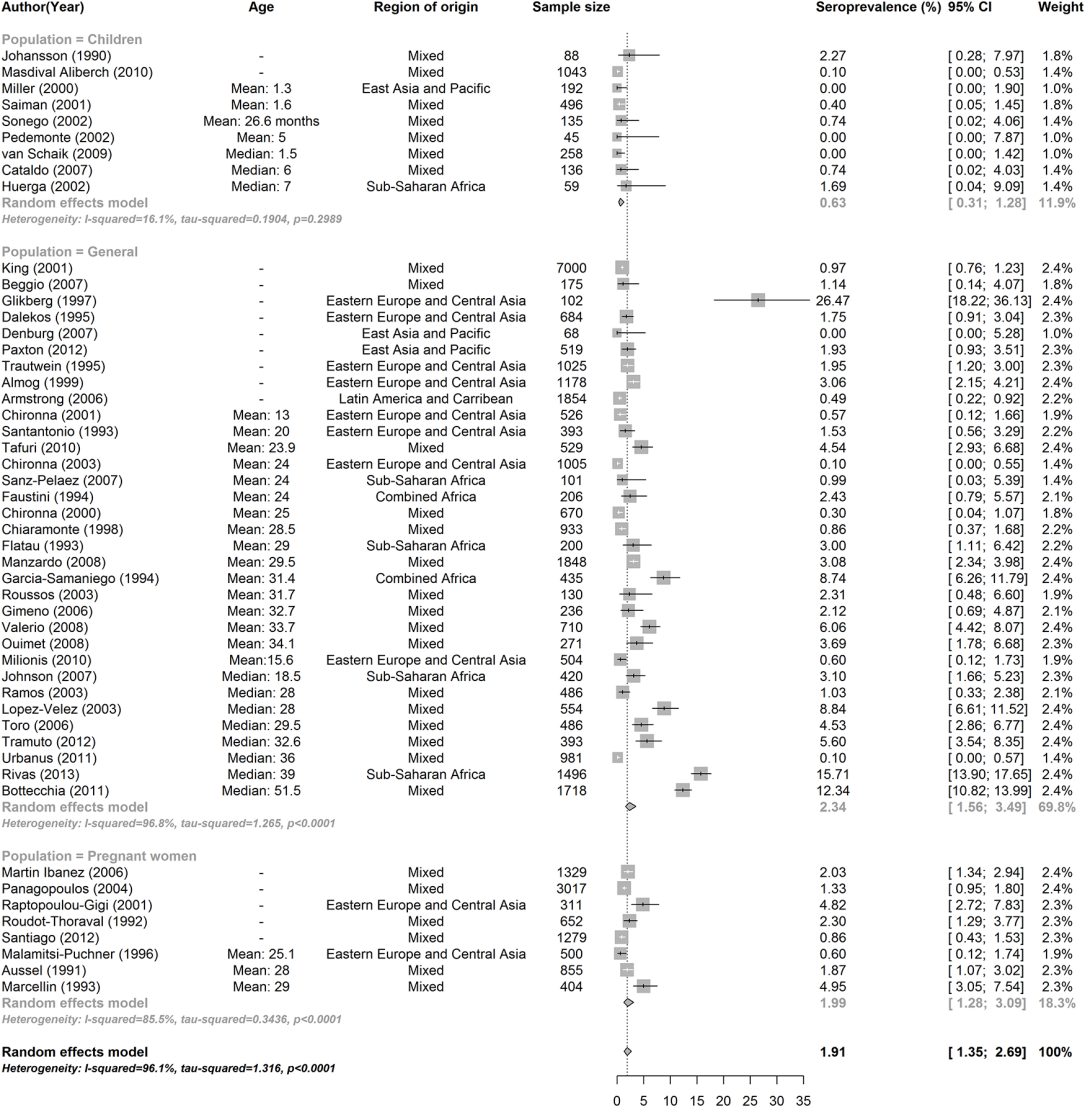
HCV seroprevalence for all included studies stratified by population group and sorted by year of publication (Forest Plot). Regions of Origin: Latin America & Caribbean (LAC), Eastern Europe & Central Asia (ECA), Middle East & North Africa (MENA), Sub-Saharan Africa (SSA), South Asia (SA), East Asia & Pacific (EAP), Mixed (a single study reporting seroprevalence from multiple regions of origin).

**Fig 3:**
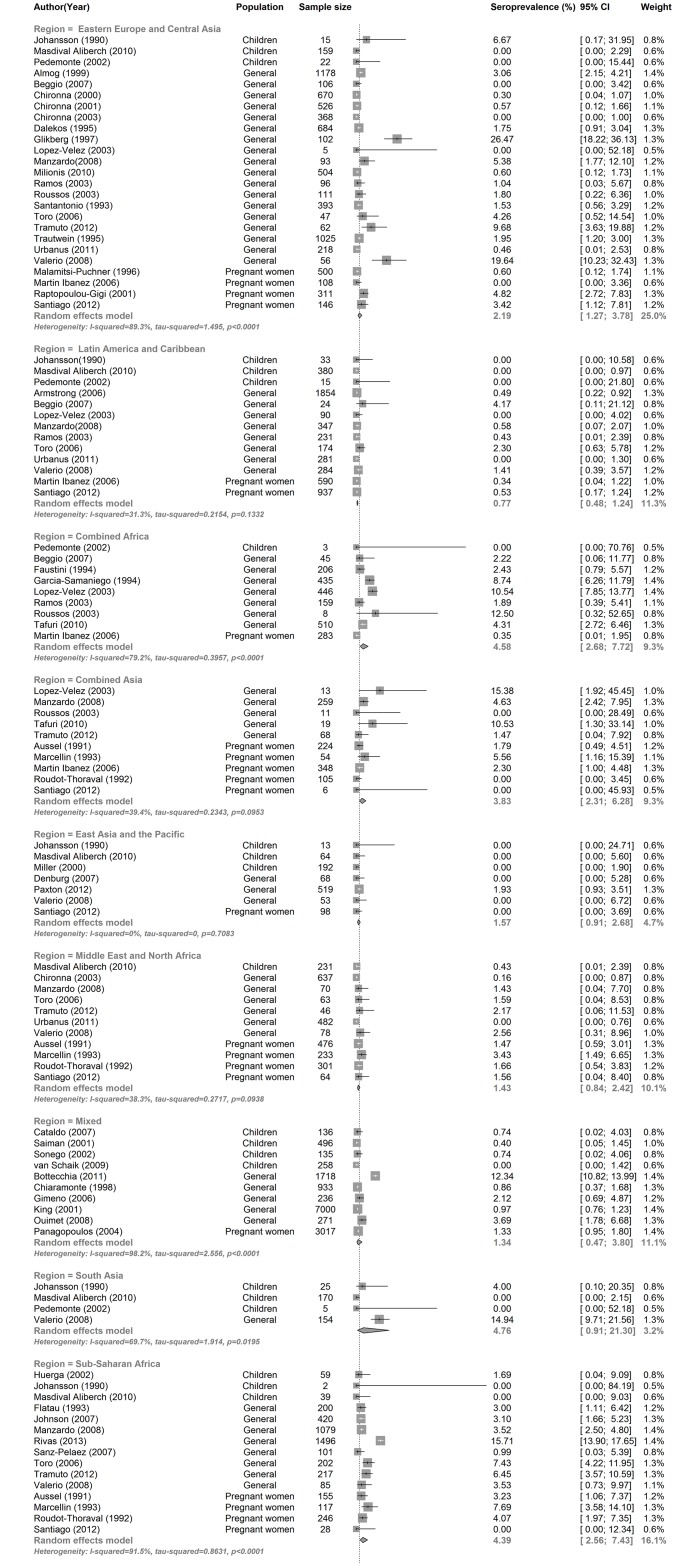
HCV seroprevalence for all included studies stratified by migrants region of origin (Forest Plot).

**Fig 4:**
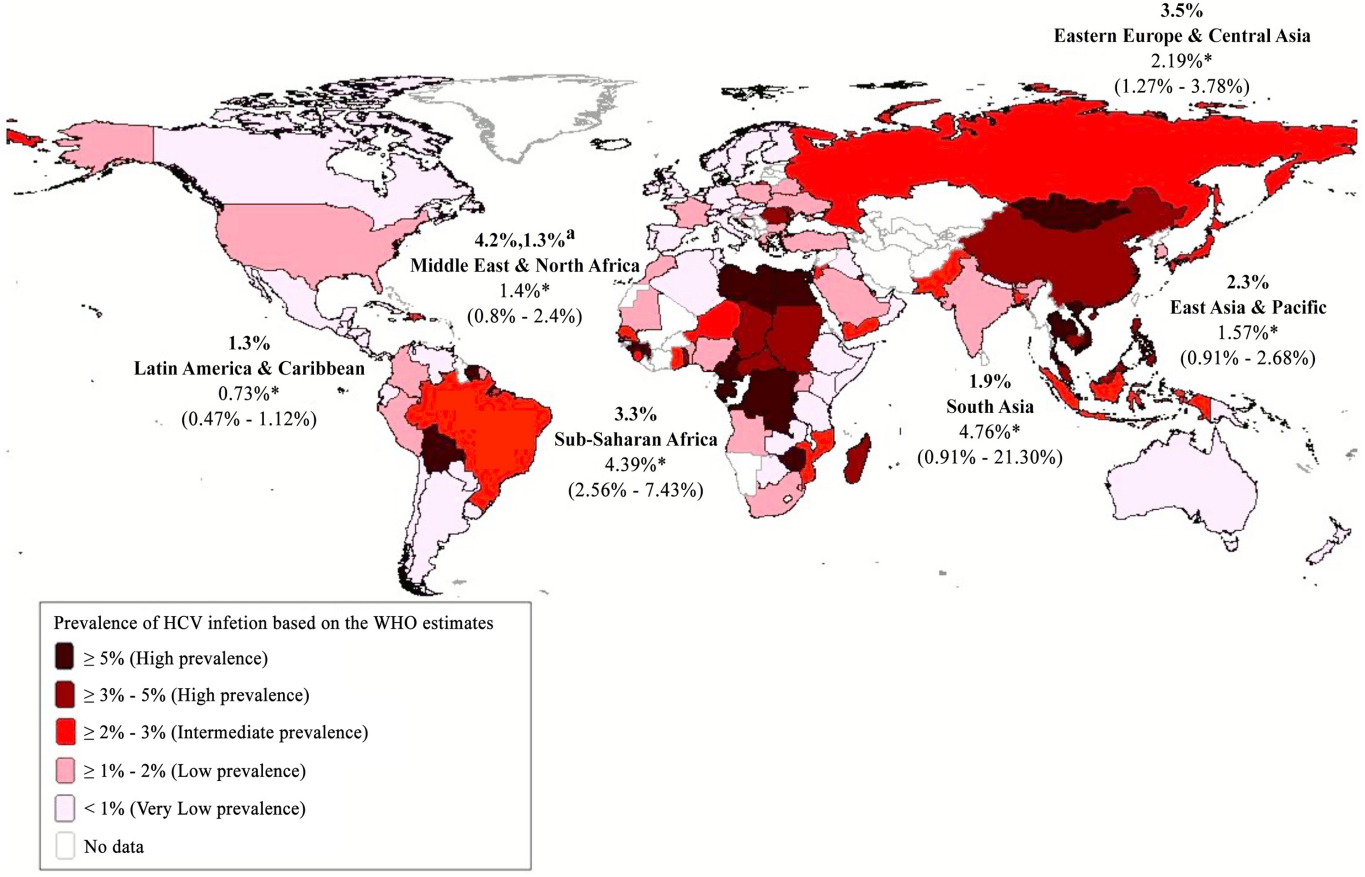
Anti-HCV seroprevalence stratified by region of origin (Map). Weighted regional HCV estimates (in bold) were based on country specific seroprevalence from the WHO or other more recent data (Table A in S4 Appendix) and 2009 country population estimates from the World Bank. ^a^ Seroprevalence excluding Egypt. * Estimates from our random-effects meta-analysis.

Age was a significant predictor of HCV seroprevalence in the 35 of 50 studies that reported the age of the studied population (Table 2, S1 Fig). Individuals in the ≥ 30 age group were more likely to have HCV antibodies as compared to those ≤18 years of age [5.6% (95% CI, 3.7–8.5% vs. 0.8% (95% CI, 0.4–1.6)] and an adjusted meta-regression model found a 8.6 (95% CI, 3.0–24.7) increased odds of being HCV positive after adjusting for migration status (Table 2). In addition, the anti-HCV prevalence in studies that only included children was significantly lower than the seroprevalence in the general population [0.6% (95% CI, 0.3–1.3) vs. 2.3% (95% CI, 1.6–3.5)] (Table 2, Fig 2). When studies that only included children were removed from the analysis, the overall anti-HCV prevalence increased from 1.9% (95% CI, 1.4–2.7) to 2.2% (95% CI, 1.6–3.2). Anti-HCV prevalence also varied significantly by region of origin, and was high (>3%) in migrants from South Asia, Sub-Saharan Africa, combined Africa and combined Asia, intermediate (2–3%) in migrants from Eastern Europe, and low (< 2%) in migrants from the Middle East & North Africa, East Asia & the Pacific, and Latin America and the Caribbean (Table 2, Fig 3). Significant differences in the seroprevalence between regions of origin remained in the meta-regression model even after adjusting for migration status (data not shown). The regional HCV seroprevalence estimates for migrants from our meta-analysis were similar or slightly lower than the corresponding HCV seroprevalence estimates from the WHO in the general population in their regions of origin with the exception of Sub-Saharan Africa and South Asia (Table 2, Fig 4). Calendar time did not affect our results as anti-HCV prevalence was similar across all decades and heterogeneity within groups remained high.

## Discussion

The findings of this study suggest that migrants from intermediate or high HCV prevalence countries represent an important risk group for HCV infection. The overall pooled anti-HCV seroprevalence in migrants in this study was 1.9% (1.4%-2.7%). Those from Sub-Saharan Africa, Asia and Eastern Europe and older age groups had the highest risk with anti-HCV prevalence ranging from 2.2% to 5.6%.

The regional HCV seroprevalence estimates from our study were a little lower or higher but fell within a similar range and pattern when compared to published studies from migrant source countries [1–3, 32–35]. Globally, anti-HCV prevalence is highest in Asia, Africa, Eastern Europe, and North Africa & the Middle East, ranging from 2–4%, whereas in North America, Latin American & the Caribbean, most Western European countries and Australia, the anti-HCV prevalence is less than 1.5% [3, 30–35]. We also found that anti-HCV seroprevalence increased with age and was higher in adults as compared to children. The similar age trend and the pattern and rank of regional estimates provide external validity to our results.

Despite similarities to published regional anti-HCV seroprevalence, the heterogeneity of the overall pooled estimate from our study was high and remained moderate even after stratification by important HCV predictive variables such as age and region of origin. The high overall and residual heterogeneity after stratification may reflect; 1) the true variability of HCV epidemiology within countries and regions, 2) characteristics of migrants included in our study and/or 3) the inability to adequately adjust for age and country of origin due the fact that data were not stratified by both variables in the studies included.

HCV epidemiology is highly variable both within and between countries and regions of origin. HCV is unevenly distributed in most countries and is usually concentrated in certain risk groups or birth cohorts and may change over time as affected cohorts grow older. The prevalence of HCV in any given country is determined by the calendar time the virus was introduced into the country, when or if blood screening is performed, and the ongoing risk of HCV exposure [2, 36]. Age is also an important predictor of HCV infection and increases with age in most settings [2]. Regional HCV prevalence may be heterogeneous due to the wide range of anti-HCV prevalence of individual countries within the same region [34, 35, 37]. The most striking example is the Middle East & North Africa which contains both Egypt with very high anti-HCV prevalence (>14%) and Djibouti, which has a low prevalence (<1%). Our regional estimates may therefore have been influenced by the number of migrants from high burden countries such as Egypt in the North African region or Pakistan in the South Asian region. We could neither adjust for this nor estimate how much this may have affected the study results. Calendar time did not affect our results as anti-HCV prevalence was similar across all decades and heterogeneity within groups remained high.

The regional anti-HCV estimates in our study were slightly lower or higher than those reported in source countries. This may be due to the fact that the migrants included in our review may have differed from the populations in their countries of origin in several ways. First, the risk profile of the two populations may be different. The majority of country level HCV estimates are based on data extrapolated from or modeled from data obtained from adult groups that are either at low or high HCV risk, whereas these populations were excluded in our study [10, 31–35, 37, 38]. Second, the inclusion of children in our study may have decreased our overall rate compared to reported rates. When children were excluded from our sample, the overall prevalence increased from 1.9% to 2.2%, an estimate closer to global estimates [1–3]. Finally, migrants may have truly lower HCV rates compared to those living in source countries due to the “healthy immigrant” effect. Many migrants, excluding refugees, are self-selected and have a higher socio-economic status (SES) and higher levels of education as compared to those living in their countries of origin [39].

The data included in this review has a number of limitations. Age was missing in 50% of study subjects and data in each region of origin was not stratified by age. We were therefore unable to adjust for both age and region of origin in the meta-regression and this may explain some of the residual heterogeneity despite stratification by these variables. There were very few study subjects from certain geographic regions such as South Asia leading to low precision in this group. Measurement bias is possible but unlikely had a large impact on the accuracy of our results. This is because the majority of studies (82.6%) used a third generation EIA and prevalence estimates were similar for studies with and without confirmatory testing. Sources of grey literature were not systematically searched and thus some data may have been missed. Finally, given the small number of studies (N = 6 accounting for only 13% of the study population) reporting HCV PCR we were not able to estimate the number of viremic HCV cases in migrants that could benefit from antiviral therapy. Despite these limitations and the uncertainty regarding all the source of the heterogeneity of our study results, it is both plausible and likely that immigrants from intermediate and high HCV endemic countries have an HCV prevalence that is similar or slightly lower than that in their source countries. The strength of this study is that it included a large number of migrants over four decades arriving in all major immigrant receiving regions.

Untreated HCV is an enormous health and economic burden that is projected to increase over the next decade unless asymptomatic infected individuals are detected and treated prior to developing end stage liver disease [6]. Eradication of HCV is now within reach given the recent availability of highly effective, well tolerated, short course, direct acting anti-viral treatments [9, 40]. Identifying and treating all individuals with HCV infection therefore, is more important than ever in order to decrease the rising economic and individual burden from HCV.

The majority of migrants (>70%) received in Canada and most European countries originate from intermediate and high HCV prevalence countries [41–62]. Recent studies show that European migrants make up a disproportionate number of HCV cases and have a higher prevalence of HCV compared to host populations [32, 37]. Similarly in Canada, migrants were estimated to accounted for 20% of HCV cases in 2002 [63]. Migrants generally lack traditional HCV risk behaviors given that they are most likely to have been infected in their countries of origin through contaminated needles, procedures and blood products [64]. Neither the risk factor based program, which detects <50% of HCV cases or the new CDC birth cohort screening program will detect HCV in most migrants [10]. Despite the well-known differences in global distribution of HCV and the potential increased risk of HCV in many migrants, only the UK has recently recommended HCV screening in migrants who were born in countries with intermediate or high seroprevalence of HCV antibodies (2% or greater).

Adult migrants originating from intermediate and high HCV prevalence regions such as Africa, Asia and Eastern Europe are an ideal additional group to consider for targeted HCV screening. Given the limitations of our study, screening would serve, at least, to provide robust seroprevalence and viremic data at the country level to identify which migrants have the highest HCV infection rates. Several studies have shown that it is cost effective to screen the general population at an anti-HCV prevalence of 3% with older interferon based therapies [65, 66]. The cost-effectiveness of screening and treatment with new highly effective but costly medications is still to be determined [67, 68]. Cost-effectiveness analysis of HCV screening in migrants would help guide the decision as to which migrants would benefit the most from screening for HCV.

The recent revolution in HCV treatment provides promise to cure all infected patients. Recent studies have shown that HCV can be eliminated in the next 15–20 years with focused strategies to screen and cure current infection as well as prevent new infections [40, 69]. Additional groups at increased HCV risk will need to be identified and treated if this potential is to be realized. In the current HCV context, the results of our study suggest that adult migrants originating from intermediate or high endemic countries are at high risk for HCV and may benefit from targeted HCV screening.

## Supporting Information

S1 Appendix PRISMA Checklist. (DOC)

S2 Appendix Search Strategy in Electronic Databases. (DOCX)

S3 Appendix Quality Assessment of Included Studies. (DOCX)

S4 Appendix Country specific estimate. (DOCX)

S1 Fig Forest plot of seroprevalence by age groups. (TIFF)

## References

[1] Lavanchy D. Evolving epidemiology of hepatitis C virus. Clin Microbiol Infect. 2011;17(2):107–15. 10.1111/j.1469-0691.2010.03432.x 21091831

[2] Hanafiah KM, Groeger J, Flaxman AD, Wiersma ST. Global epidemiology of hepatitis C virus infection: new estimates of age-specific antibody to HCV seroprevalence. Hepatology. 2013;57(4):1333–42. 10.1002/hep.26141 23172780

[3] Gower E, Estes CC, Hindman S, Razavi-Shearer K, Razavi H. Global epidemiology and genotype distribution of the hepatitis C virus infection. J Hepatol. 2014;61(Suppl. 1):S45–57. 25086286 10.1016/j.jhep.2014.07.027

[4] Perz JF, Armstrong GL, Farrington LA, Hutin YJ, Bell BP. The contributions of hepatitis B virus and hepatitis C virus infections to cirrhosis and primary liver cancer worldwide. J Hepatol. 2006;45(4):529–38. 16879891 10.1016/j.jhep.2006.05.013

[5] Nordenstedt H, White DL, El-Serag HB. The changing pattern of epidemiology in hepatocellular carcinoma. Digest Liver Dis. 2010;42 Suppl 3:S206–14. 10.1016/S1590-8658(10)60507-5 PMC3392755 20547305

[6] El Khoury AC, Wallace C, Klimack WK, Razavi H. Economic burden of hepatitis C-associated diseases: Europe, Asia Pacific, and the Americas. J Med Econ. 2012;15(5):887–96. 10.3111/13696998.2012.681332 22458755

[7] Hajarizadeh B, Grebely J, Dore GJ. Epidemiology and natural history of HCV infection. Nat Rev Gastroentero. 2013;10(9):553–62. 10.1038/nrgastro.2013.107 23817321

[8] Lam BP, Jeffers T, Younoszai Z, Fazel Y, Younossi ZM. The changing landscape of hepatitis C virus therapy: focus on interferon-free treatment. Therap Advan Gastroenterol. 2015;8(5):298–312. 10.1177/1756283X15587481 PMC4530432 26327920

[9] Feeney ER, Chung RT. Antiviral treatment of hepatitis C. BMJ. 2014;349:g3308. 10.1136/bmj.g3308 PMC6880951 25002352

[10] Smith BD, Morgan RL, Beckett GA, Falck-Ytter Y, Holtzman D, Ward JW. Hepatitis C Virus Testing of Persons Born During 1945 to 1965: Recommendations From the Centers for Disease Control and Prevention. Ann Intern Med. 2012. 10.7326/0003-4819-157-9-201211060-00529 PMC5777166 22910836

[11] Trubnikov M, Yan P, Archibald C. Estimated prevalence of Hepatitis C Virus infection in Canada, 2011. Canada Communicable Disease Report (CCDR) 2014;40–19:421–9. 10.14745/ccdr.v40i19a02 PMC5864479 29769874

[12] Moyer VA. Screening for hepatitis C virus infection in adults: US Preventive Services Task Force recommendation statement. Ann Intern Med. 2013;159(5):349–57. 10.7326/0003-4819-159-5-201309030-00672 23798026

[13] Greenaway C, Wong D, Assayag D, Deschenes M, Hui C, Ueffing E, et al Hepatitis C: Evidence-based clinical guidelines for immigrants and refugees. Can Med Assoc J. 2011;183(12):E861–E4. 10.1503/cmaj.090313 PMC3168666 20530168

[14] Pepin J, Abou Chakra CN, Pepin E, Nault V, Valiquette L. Evolution of the global burden of viral infections from unsafe medical injections, 2000–2010. PloS one. 2014;9(6):e99677 10.1371/journal.pone.0099677 24911341 PMC4049770

[15] National Institute for Health and Clinical Excellence. Hepatitsi B and C- ways to promote and offer testing 2012. Accessed 10 August 2014. Available: http://www.nice.org.uk/guidance/ph43.

[16] Liberati A, Altman D, Tetzlaff J, Mulrow C, Gotzsche P, Ioannidis J, et al The PRISMA Statment for Reporting Systematic Reviews and Meta-Analyses of Studies that Evaluate Health Care Interventions: Explanation and Elaboration. Annals Inter Med. 2009;152(4):W65–W94. 10.7326/0003-4819-151-4-200908180-00136 19622512

[17] Guyatt G, Oxman AD, Akl EA, Kunz R, Vist G, Brozek J, et al GRADE guidelines: 1. Introduction-GRADE evidence profiles and summary of findings tables. J Clin Epidemiol. 2011;64(4):383–94. 10.1016/j.jclinepi.2010.04.026 21195583

[18] Iorio A, Spencer FA, Falavigna M, Alba C, Lang E, Burnand B, et al Use of GRADE for assessment of evidence about prognosis: rating confidence in estimates of event rates in broad categories of patients. BMJ. 2015;350:h870 10.1136/bmj.h870 25775931

[19] Hunter J, Saratzis A, Sutton A, Boucher R, Sayer R, Bown M. In meta-analyses of proprtion studies, funnel plots were found to be an inaccurate method of assessing publication bias. J Clin Epidemiol. 2014;67:897–903. 10.1016/j.jclinepi.2014.03.003 24794697

[20] Slot E, Janssen MP, Marijt-van der Kreek T, Zaaijer HL, van de Laar TJ. Two decades of risk factors and transfusion-transmissible infections in Dutch blood donors. Transfusion. 2015. 10.1111/trf.13298 26355711

[21] Murphy EL, Fang J, Tu Y, Cable R, Hillyer CD, Sacher R, et al Hepatitis C virus prevalence and clearance among US blood donors, 2006–2007: associations with birth cohort, multiple pregnancies, and body mass index. J Infect Dis. 2010;202(4):576–84. 10.1086/654882 20617929 PMC2932782

[22] Mitrovic N, Delic D, Markovic-Denic L, Jovicic M, Popovic N, Bojovic K, et al Seroprevalence and risk factors for hepatitis C virus infection among blood donors in Serbia: A multicentre study. Dig Liver Dis. 2015;47(7):572–6. 10.1016/j.dld.2015.03.019 25882212

[23] The World Bank. Countries and Economies 2014. Accessed 13 January 2014. Available: http://data.worldbank.org/country.

[24] Fleiss JL. Review papers: The statistical basis of meta-analysis. Stat Methods Med Res. 1993;2(2):121–45. 8261254 10.1177/096228029300200202

[25] Barendregt JJ, Doi SA, Lee YY, Norman RE, Vos T. Meta-analysis of prevalence. J Epidemiol Commun H. 2013;67(11):974–8. 10.1136/jech-2013-203104 23963506

[26] Rücker G, Schwarzer G, Carpenter J, Olkin I. Why add anything to nothing? The arcsine difference as a measure of treatment effect in meta-analysis with zero cells. Stat Med. 2009;28(5):721–38. 10.1002/sim.3511 19072749

[27] Higgins J, Thompson SG. Quantifying heterogeneity in a meta-analysis. Stat Med. 2002;21(11):1539–58. 12111919 10.1002/sim.1186

[28] Schwarzer G. Meta: Meta-Analysis with R. Available: http://cran.r-project.org/web/packages/meta/.

[29] Bates D, Maechler M, Bolker B. lme4: Linear mixed-effects models using S4 classes. 2012.

[30] World Health Organization. Hepatitis C—global prevalence (Update). Wkly Epidemiol Rec. 1999;74(49):425–7. 10645164

[31] Cornberg M, Razavi HA, Alberti A, Bernasconi E, Buti M, Cooper C, et al A systematic review of hepatitis C virus epidemiology in Europe, Canada and Israel. Liver Int. 2011;31 Suppl 2:30–60. 10.1111/j.1478-3231.2011.02539.x 21651702

[32] European Centre for Disease Prevention and Control. Hepatitis B and C in the EU neighbourhood: prevalence, burden of disease and screening policies. Stockholm: ECDC, 2010.

[33] Kershenobich D, Razavi HA, Sanchez-Avila JF, Bessone F, Coelho HS, Dagher L, et al Trends and projections of hepatitis C virus epidemiology in Latin America. Liver Int. 2011;31(Suppl 2):18–29. 21651701 10.1111/j.1478-3231.2011.02538.x

[34] Sievert W, Altraif I, Razavi HA, Abdo A, Ahmed EA, Alomair A, et al A systematic review of hepatitis C virus epidemiology in Asia, Australia and Egypt. Liver Int. 2011;31(SUPPL. 2):61–80. 10.1111/j.1478-3231.2011.02540.x 21651703

[35] Madhava V, Burgess C, Drucker E. Epidemiology of chronic hepatitis C virus infection in sub-Saharan Africa. Lancet Infect Dis. 2002;2(5):293–302. 12062995 10.1016/s1473-3099(02)00264-5

[36] Alter MJ. Epidemiology of hepatitis C virus infection. World J Gastroenterol 2007;13(17):2436–41. 17552026 10.3748/wjg.v13.i17.2436 PMC4146761

[37] Hatzakis A, Damme P, Alcorn K, Gore C, Benazzouz M, Berkane S, et al The state of hepatitis B and C in the Mediterranean and Balkan countries: report from a summit conference. J Viral Hepatitis. 2013;20(s2):1–20. 10.1111/jvh.12120 23827008

[38] Remis RS. Modelling the incidence and prevalence of hepatitis C infection and its sequelae in Canada, 2007. Public Health Agency of Canada website [updated 2007. 2011:46.

[39] Greenaway C, Dongier P, Boivin J-F, Tapiero B, Miller M, Schwartzman K. Susceptibility to measles, mumps, and rubella in newly arrived adult immigrants and refugees. Ann Intern Med. 2007;146(1):20–4. 17200218 10.7326/0003-4819-146-1-200701020-00005

[40] Wedemeyer H, Duberg AS, Buti M, Rosenberg WM, Frankova S, Esmat G, et al Strategies to manage hepatitis C virus (HCV) disease burden. J Viral Hepatitis. 2014;21:60–89. 10.1111/jvh.12249 24713006

[41] Statistics Canada. Immigrant Status and Period of Immigration and Place of Birth for the Immigrants and Non-permanent Residents of Canada, Provinces, Territories, Census Metropolitan Areas and Census Agglomerations, 2006 Census: Statistics Canada; 2006. Available: http://www12.statcan.gc.ca/census-recensement/2006/rt-td/index-eng.cfm.

[42] U.S. Census Bureau. American Community Survey. Tables S0501 –S0506. Selected Characteristics of the Foreign-Born Population by Region of Birth: U.S. Census Bureau; 2009. Accessed 1 August 2011. Available: http://www.census.gov/acs/www/.

[43] Statistik Austria. Bevolkerungsstand 2010—Population by Citizenship (Table 7) Vienna: Statistik Austria; 2010 Accessed 1 August 2011. Available: http://www.statistik.at/dynamic/wcmsprod/idcplg?IdcService=GET_NATIVE_FILE&dID=85783&dDocName=053629.

[44] Statistics Belgium. Population par nationalité, sexe, groupe et classe d'âges au 1er janvier 2010: Statistics Belgium; 2011. Accessed 20 August 2011. Available: http://statbel.fgov.be/fr/modules/publications/statistiques/population/population_natio_sexe_groupe_classe_d_ges_au_1er_janvier_2010.jsp.

[45] Czech Statistical Office. Foreigners in the Czech Republic, 2010: Czech Statistical Office; 2010. Accessed 22 August 2011. Available: http://www.czso.cz/csu/2010edicniplan.nsf/engp/1414-10.

[46] Statistics Denmark. Population by ancestry, region, time, citizenship, age, country of origin and sex: Statistics Denmark; 2009. 19 August 2011. Available: http://www.statbank.dk/statbank5a/default.asp?w=1280.

[47] Statistics Finland. Country of birth according to age and gender by region 1990–2010: Statistics Finland; 2011. Accessed 22 August 2011. Available: http://pxweb2.stat.fi/database/StatFin/vrm/vaerak/vaerak_en.asp.

[48] Institut national de la statistique et des études économiques. Recensement de la population—Étrangers selon le sexe, la catégorie de population et la nationalité détaillée Paris: Institut national de la statistique et des études économiques; 2006 Accessed 22 August 2011. Available: http://www.insee.fr/fr/bases-de-donnees/default.asp?page=recensements.htm.

[49] Statistisches Bundesamt. Statistical Yearbook 2010—Table 2.20: Statistisches Bundesamt; 2010. Accessed 1 September 2011. Available: http://www.destatis.de/jetspeed/portal/cms/Sites/destatis/Internet/EN/Navigation/Publications/Crosssection/Yearbook.psml.

[50] Observatory MM. Data on immigrants in Greece, from Census 2001, Legalization applications 1998, and valid Residence Permits, 2004—Table A1: Mediterranean Migration Observatory; 2004. Accessed 24 August 2011. Available: http://www.mmo.gr/statistics_greece.htm.

[51] Israel Central Bureau of Statistics. 2008 Census: Males and Females born abroad by continent of birth, country of birth and age group—Tables 1–10 and 1–11: Israel Central Bureau of Statistics; 2008. Accessed 14 September 2011. Available: http://www1.cbs.gov.il/census/census/pnimi_page_e.html?id_topic=11

[52] Caritas Italiana. Dossier Statistico Immigrazione Caritas-Migrantes 2010 Rome: Caritas Italiana; 2010 Accessed 20 August 2011. Available: http://www.dossierimmigrazione.it/.

[53] Statistics Netherlands. Population in The Netherlands on 1 January 2011 by sex, age, marital status, origin and generation. The Hague: Statistics Netherlands; 2011 Accessed 22 August 2011. Available: http://statline.cbs.nl/StatWeb/publication/?VW=T&DM=SLEN&PA=37325eng&LA=EN.

[54] Statistics Norway. Table 05185—Foreign born, by sex and country background: Statistics Norway; 2011. Accessed 19 August 2011. Available: http://statbank.ssb.no/statistikkbanken/Default_FR.asp?PXSid=0&nvl=true&PLanguage=1&tilside=selectvarval/define.asp&Tabellid=05185.

[55] Servico de Estrangeiros e Fronteiras. Relatorio de Imigracao Fronteiras e Asilo: Servico de Estrangeiros e Fronteiras; 2010. Accessed 19 August 2011. Available: www.sef.pt.

[56] Ireland CSO. Population usually resident (and present in their usual residence on census night) in each Province and in the Aggregate Town and Aggregate Rural Areas, classified by birthplace: Central Statistics Office Ireland; 2006. Accessed 26 September 2011. Available: http://www.cso.ie/en/census/census2006reports/census2006volume4-usualresidencemigrationbirthplacesandnationalities/.

[57] Estadística INd. National Immigrant Survey—Revisión del Padrón municipal 2007. Datos a nivel nacional, comunidad autónoma y provincia.: Instituto Nacional de Estadística; 2008. Accessed 19 August 2011. Available: http://www.ine.es/en/inebmenu/mnu_migrac_en.htm.

[58] Statistics Sweden. Foreign-born persons in Sweden by country of birth and sex.: Statistics Sweden; 2011. Accessed 22 August 2011. Available: http://www.ssd.scb.se/databaser/makro/MainTable.asp?yp=bergman&xu=scb&omradekod=BE&omradetext=Population&lang=2&langdb=2.

[59] Swiss Federal Institute of Statistics. Population résidante selon la nationalité par pays, de 1995 à 2009: Swiss Federal Institute of Statistics; 2010. Accessed 1 August 2011. Available: http://www.bfs.admin.ch/bfs/portal/fr/index/themen/01/07/blank/data/01.html.

[60] BBC News. BBC News. Born Abroad: An Immigration Map of Britain 2008. Accessed 19 August 2011. Available: http://news.bbc.co.uk/2/shared/spl/hi/uk/05/born_abroad/countries/html/overview.stm.

[61] Australian Bureau of Statistics 2006 Census of Population and Housing—Country of Birth of Person (full classification list) by Sex—Catalogue 2068.0 Canberra: Australian Bureau of Statistics; 2007 Accessed 8 August 2011. Available: http://www.abs.gov.au/websitedbs/d3310114.nsf/home/census+data.

[62] Statistics New Zealand. 2006 Census of Population and Housing: QuickStats About Culture and Identity, Table 7: Statistics New Zealand; 2006. Accessed 3 August 2011. Available: http://www.stats.govt.nz/Census/2006CensusHomePage/QuickStats/quickstats-about-a-subject/culture-and-identity.aspx.

[63] Remis RS. A Study to Characterize the Epidemiology of Hepatitis C Infection in Canada, 2002. Final Report. Public Health Agency of Canada. 2004.

[64] Asselah T, Perumalswami P, Dieterich D. Is Screening Baby Boomers for HCV Enough? A Call to Screen for Hepatitis C Virus in Persons from Countries of High Endemicity. Liver Int. 2014;34(10):1447–51. 10.1111/liv.12599 24840955

[65] Coffin PO, Scott JD, Golden MR, Sullivan SD. Cost-effectiveness and population outcomes of general population screening for hepatitis C. Clin Infect Dis. 2012;54(9):1259–71. 10.1093/cid/cis011 22412061 PMC3404694

[66] Rein DB, Smith BD, Wittenborn JS, Lesesne SB, Wagner LD, Roblin DW, et al The cost-effectiveness of birth-cohort screening for hepatitis C antibody in U.S. primary care settings. Ann Inter Med. 2012;156(4):263–70. 10.7326/0003-4819-156-4-201202210-00378 PMC5484577 22056542

[67] Hagan L, Sulkowski M, Schinazi R. Cost analysis of sofosbuvir/ribavirin versus sofosbuvir/simeprevir for genotype 1 hepatitis C virus in interferon ineligibe/intolerant individuals. Hepatology. 2014;60(1):37–45. 10.1002/hep.27151 24677184 PMC4077973

[68] Pho M, Linas M. Valuing Cure: Bridging Cost-Effectiveness adn Coverage Decision for Hepatitis C Therapy. Hepatology. 2014;60(1):12–4. 10.1002/hep.27220 24825115 PMC4096113

[69] Razavi H, Waked I, Sarrazin C, Myers RP, Idilman R, Calinas F, et al The present and future disease burden of hepatitis C virus (HCV) infection with today's treatment paradigm. J Viral Hepatitis. 2014;21:34–59. 10.1111/jvh.12248 24713005

[70] Almog R, Low M, Cohen D, Robin G, Ashkenazi S, Bercovier H, et al Prevalence of anti-hepatitis A antibodies, hepatitis B viral markers, and anti-hepatitis C antibodies among immigrants from the former USSR who arrived in Israel during 1990–1991. Infection. 1999;27(3):212–7. 10378135 10.1007/BF02561531

[71] Armstrong GL, Wasley A, Simard EP, McQuillan GM, Kuhnert WL, Alter MJ. The prevalence of hepatitis C virus infection in the United States, 1999 through 2002. Ann Intern Med. 2006;144(10):705–14. 16702586 10.7326/0003-4819-144-10-200605160-00004

[72] Aussel L, Denis F, Ranger S, Martin P, Caillaud M, Alain J, et al Prevalence of antibodies to the hepatitis C virus in pregnant foreign residents in France. Pathol Biol. 1991;39(10):991–6. 1805141

[73] Beggio M, Giraldo M, Borella-Venturini M, Mongillo M, Zanetti E, Bruno A, et al Prevalence of hepatitis virus A, B, and C markers according to the geographic origin of medical students. G Ital Med Lav Ergon. 2007;29(3):745–7. 18409937

[74] Bottecchia M, Madejon A, Puente S, Garcia-Samaniego J, Rivas P, Herrero D, et al Detection of hepatitis B virus genotype A3 and primary drug resistance mutations in African immigrants with chronic hepatitis B in Spain. J Antimicrob Chemoth. 2011;66(3):641–4. 10.1093/jac/dkq484 21177673

[75] Cataldo F, Viviano E. Health problems of internationally adopted children. Ital J Pediatr. 2007;33(2):92–9.

[76] Chiaramonte M, Pupo A, Menegon T, Baldo V, Malatesta R, Trivello R. HBV and HCV infection among non-European Union immigrants in North-East Italy. Epidemiol Infect. 1998;121(1):179–83. 9747770 10.1017/s0950268898001034 PMC2809489

[77] Chironna M, Germinario C, Lopalco PL, Carrozzini F, Barbuti S, Quarto M. Prevalence rates of viral hepatitis infections in refugee Kurds from Iraq and Turkey. Infection. 2003;31(2):70–4. 12682810 10.1007/s15010-002-3100-3

[78] Chironna M, Germinario C, Lopalco PL, Quarto M, Barbuti S. HBV, HCV and HDV infections in Albanian refugees in Southern Italy (Apulia region). Epidemiol Infect. 2000;125(1):163–7. 11057972 10.1017/s0950268899004215 PMC2869582

[79] Chironna M, Germinario C, Lupalco PL, Carrozzini F, Quarto M. Prevalence of hepatitis virus infections in Kosovar refugees. Int J Infect Dis. 2001;5(4):209–13. 11953219 10.1016/s1201-9712(01)90073-6

[80] Dalekos GN, Zervou E, Karabini F, Tsianos EV. Prevalence of viral markers among refugees from southern Albania: Increased incidence of infection with hepatitis A, B and D viruses. Eur J Gastroenterol Hepatol. 1995;7(6):553–8. 7552639

[81] Denburg A, Rashid M, Brophy J, Curtis T, Malloy P, Audley J, et al Initial health screening results for Karen refugees: a retrospective review. CCDR. 2007;33(13):16–22. 18161207

[82] Faustini A, Franco E, Saitto C, Cauletti M, Zaratti L, Papini P, et al Hepatitis A, B, C and D in a community in Italy of immigrants from NE Africa. J Public Health Med. 1994;16(1):71–8. 8037956 10.1093/oxfordjournals.pubmed.a042938

[83] Flatau E, Segol O, Shneour A, Tabenkin H, Raz R. Prevalence of markers of infection with hepatitis B and C viruses in immigrants of operation Solomon, 1991. Israel J Med Sci. 1993;29(6–7):387–9. 8394300

[84] Garcia-Samaniego J, Soriano V, Enriquez A, Lago M, Martinez ML, Muno F. Hepatitis B and C virus infections among African immigrants in Spain. Am J Gastroenterol. 1994;89(10):1918–9. 7942709

[85] Gimeno LA, Fernandez De Alarcon Medina I, Perez Alonso E, Penalva D, Ruiz-Gimenez Aguilar JL, Ramos EM. Health examination of immigrants in Primary Care. Enferm Emerg. 2006;8(1):40–7.

[86] Glikberg F, Brawer-Ostrovsky J, Ackerman Z. Very high prevalence of hepatitis B and C in Bukharian jewish immigrants to israel. J Clin Gastroenterol. 1997;24(1):30–3. 9013347 10.1097/00004836-199701000-00006

[87] Huerga H, Lopez-velez R. Infectious diseases in sub-Saharan African immigrant children in Madrid, Spain. Pediatr Infect Dis J. 2002;21(9):830–4. 12352804 10.1097/01.inf.0000027667.32707.dd

[88] Johansson PJH, Lofgren B, Nordenfelt E. Low frequency of hepatitis C antibodies among children from foreign countries adopted in Swedish families. Scand J Infect Dis. 1990;22(5):619–20. 2175449 10.3109/00365549009027106

[89] Johnson D. Rates of infectious disease and nutritional deficiencies in newly arrived African refugees. Government of South Australia, Central North Adelaide Health Service. 2007.

[90] King K, Vodicka P. Screening for conditions of public health importance in people arriving in Australia by boat without authority. Med J Aust. 2001;175(11–12):600–2. 11837856 10.5694/j.1326-5377.2001.tb143742.x

[91] Lopez-Velez RPD, Turrientes CMD, Gutierrez CMD, Mateos MMD. Prevalence of Hepatitis B, C, and D Markers in Sub-Saharan African Immigrants. J Clin Gastroenterol. 1997;25(4):650–2. 9451681 10.1097/00004836-199712000-00020

[92] Malamitsi-Puchner A, Papacharitonos S, Sotos D, Tzala L, Psichogiou M, Hatzakis A, et al Prevalence study of different hepatitis markers among pregnant Albanian refugees in Greece. Eur J Epidemiol. 1996;12(3):297–301. 8884198 10.1007/BF00145420

[93] Manzardo C, Trevino B, Gomez i Prat J, Cabezos J, Mongui E, Claveria I, et al Communicable diseases in the immigrant population attended to in a tropical medicine unit: Epidemiological aspects and public health issues. Travel Med Infect Dis. 2008;6(1–2):4–11. 10.1016/j.tmaid.2007.11.002 18342267

[94] Marcellin P, Bernuau J, Martinot-Peignoux M, Larzul D, Xu L, Tran S, et al Prevalence of hepatitis C virus infection in asymptomatic anti-HIV1 negative pregnant women and their children. Dig Dis Sci. 1993;38(12):2151–5. 8261814 10.1007/BF01299888

[95] Martin Ibanez I, Lopez Vilchez Ma A, Lozano Blasco J, Mur Sierra A. Perinatal outcomes in immigrant women. An Pediatr. 2006;64(6):550–6. 10.1157/13089920 16792963

[96] Masvidal Aliberch RM, Estabanell Buxo A, Miguel Gil B, Cruz Rodriguez C, Frutos Gallego ED, Guzman Molina C, et al Indication of determination of antibodies against hepatitis C and A viruses in the protocol for the care of young immigrants. Gac Sanit. 2010;24(4):288–92. 20053479 10.1016/j.gaceta.2009.10.013

[97] Milionis C. Serological markers of Hepatitis B and C among juvenile immigrants from Albania settled in Greece. Eur J Gen Pract. 2010;16(4):236–40. 10.3109/13814788.2010.525631 20954813

[98] Miller LC, Hendrie NW. Health of children adopted from China. Pediatrics. 2000;105(6):E76 10835089 10.1542/peds.105.6.e76

[99] Ouimet MJ, Munoz M, Narasiah L, Rambure V, Correa JA. Common diseases in asylum seekers in Montreal: Prevalence and associations with risk factors. Can J Public Health. 2008;99(6):499–504. 19149395 10.1007/BF03403785 PMC6975710

[100] Panagopoulos P, Economou A, Kasimi A, Spyropoulou P, Kanellopoulos N, Dadiotis L, et al Prevalence of hepatitis B and C in the maternity department of a Greek district hospital. J Matern Fetal Neonatal Med. 2004;16(2):106–10. 15512720 10.1080/14767050400003751

[101] Paxton GA, Sangster KJ, Maxwell EL, McBride CRJ, Drewe RH. Post-arrival health screening in Karen refugees in Australia. PloS one. 2012;7(5):e38194 10.1371/journal.pone.0038194 22693599 PMC3364970

[102] Pedemonte P, Rosso R, Iozzi C, Mantero E, Moroni C, Bassetti M, et al Infectious diseases in adopted children coming from developing countries: A possible approach. Ital J Pediatr. 2002;28(5):392–5.

[103] Ramos JM, Pastor C, Masia Ma M, Cascales E, Royo G, Gutierrez-Rodero F. Health in the immigrant population: Prevalence of latent tuberculosis, hepatitis B, hepatitis C, human immunodeficiency virus and syphilis infection. Enferm Infecc Microbiol Clin. 2003;21(10):540–2. 14642251 10.1016/s0213-005x(03)73006-7

[104] Raptopoulou-Gigi M, Orphanou E, Lalla T, Lita A, Garifallos A. Prevalence of hepatitis C virus infection in a cohort of pregnant women in northern Greece and transmission of HCV from mother to child. Eur J Epidemiol. 2001;17(3):263–6. 11680545 10.1023/a:1017951605272

[105] Rivas P. Hepatitis B, C, and D and HIV infections among immigrants from equatorial guinea living in Spain. Am J Trop Med Hyg. 2013;88(4):789–94. 10.4269/ajtmh.12-0319 23339201 PMC3617871

[106] Roudot-Thoraval F, Deforges L, Girollet PP, Maria B, Milliez J, Pathier D, et al Prevalence of hepatitis C virus antibodies (tests ELISA 2 and RIBA 2) in a population of pregnant women in France. Gastroenterol Clin Biol. 1992;16(3):255–9. 1582542

[107] Roussos A, Goritsas C, Pappas T, Spanaki M, Papadaki P, Ferti A. Prevalence of hepatitis B and C markers among refugees in Athens. World J Gastroenterol 2003;9(5):993–5. 12717844 10.3748/wjg.v9.i5.993 PMC4611411

[108] Saiman L, Aronson J, Zhou J, Gomez-Duarte C, San Gabriel P, Alonso M, et al Prevalence of infectious diseases among internationally adopted children. Pediatrics. 2001;108(3):608–12. 11533325 10.1542/peds.108.3.608

[109] Santantonio T, Lo Caputo S, Germinario C, Squarcione S, Greco D, Laddago V, et al Prevalence of hepatitis virus infections in Albanian refugees. Eur J Epidemiol. 1993;9(5):537–40. 8307139 10.1007/BF00209532

[110] Santiago B, Blazquez D, Lopez G, Sainz T, Munoz M, Alonso T, et al Serological profile of immigrant pregnant women against HIV, HBV, HCV, rubella, Toxoplasma gondii, Treponema pallidum, and Trypanosoma cruzi. Enferm Infecc Microbiol Clin. 2012;30(2):64–9. 22079225 10.1016/j.eimc.2011.07.010

[111] Sanz-Pelaez O, Santana OE, Carranza C, Perez-Arellano JL. HTLV-1 infection prevalence in recently arrived sub-Saharan immigrants. Enferm Infecc Microbiol Clin. 2007;25(9):603–7. 17953904 10.1157/13111191

[112] Sonego M, Garcia Perez J, Pereira Candel J. Health problems of foreign adopted children in Spain. Med Clin. 2002;119(13):489–91. 10.1016/s0025-7753(02)73471-4 12406396

[113] Tafuri S, Prato R, Martinelli D, Melpignano L, De Palma M, Quarto M, et al Prevalence of Hepatitis B, C, HIV and syphilis markers among refugees in Bari, Italy. BMC Infect Dis. 2010;10:213 10.1186/1471-2334-10-213 20646306 PMC2916911

[114] Toro C, Jimenez V, Rodriguez C, Del Romero J, Rodes B, Holguin A, et al Molecular and epidemiological characteristics of blood-borne virus infections among recent immigrants in Spain. J Med Virol. 2006;78(12):1599–608. 17063510 10.1002/jmv.20744

[115] Tramuto F, Mazzucco W, Maida CM, Affronti A, Affronti M, Montalto G, et al Serological pattern of Hepatitis B, C, and HIV infections among immigrants in Sicily: epidemiological aspects and implication on public health. J Community Health. 2012;37(3):547–53. 10.1007/s10900-011-9477-0 21922163

[116] Trautwein C, Kiral G, Tillmann H, Witteler H, Michel G, Manns M. Risk factors and prevalence of hepatitis E in German immigrants from the former Soviet Union. J Med Virol. 1995;45(4):429–34. 7666043 10.1002/jmv.1890450413

[117] Urbanus AT, van de Laar TJ, van den Hoek A, Zuure FR, Speksnijder AG, Baaten GG, et al Hepatitis C in the general population of various ethnic origins living in the Netherlands: should non-Western migrants be screened? J Hepatol. 2011;55(6):1207–14. Epub 2011 Apr 13. 10.1016/j.jhep.2011.02.028 21703202

[118] Valerio L, Barro S, Perez B, Roca C, Fernandez J, Solsona L, et al Seroprevalence of chronic viral hepatitis markers in 791 recent immigrants in Catalonia, Spain. Screening and vaccination against hepatitis B recommendations. Rev Clin Esp. 2008;208(9):426–31. 19000469 10.1157/13127602

[119] van Schaik R, Wolfs TF, Geelen SP. Improved general health of international adoptees, but immunization status still insufficient. Eur J Pediatr. 2009;168(9):1101–6. 10.1007/s00431-008-0895-7 19125292 PMC2714889

