# Correction: The Seroprevalence of Hepatitis C Antibodies in Immigrants and Refugees from Intermediate and High Endemic Countries: A Systematic Review and Meta-Analysis

**DOI:** 10.1371/journal.pone.0144567

**Published:** 2015-12-09

**Authors:** Christina Greenaway, Ann Thu Ma, Lorie A. Kloda, Marina Klein, Sonya Cnossen, Guido Schwarzer, Ian Shrier

S1 Fig is a duplicate of Fig 3. Please view the correct S1 Fig below.

## Supporting Information

S1 Fig Forest plot of seroprevalence by age groups. This file includes supplementary data.
(TIFF)
