# Supplementary material for: The Seroprevalence of Hepatitis C Antibodies in Immigrants and Refugees from Intermediate and High Endemic Countries: A Systematic Review and Meta-Analysis
Source: PLoS One. 2015 Nov 11;10(11):e0141715. doi: 10.1371/journal.pone.0141715 (PMC4641717; doi:10.1371/journal.pone.0141715)
Supplement: S2 Appendix — (DOCX) [file pone.0141715.s002.docx]

**Supporting Information S2**

**Search Strategy**

1. exp Hepatitis C/

2. exp Hepacivirus/

3. Hepatitis C Antigens/

4. (hepatitis c or hcv or chc or non-A or non-B).tw.

5. or/1-4 [Hepatitis C terms]

6. exp "Emigration and Immigration"/

7. exp “Emigrants and Immigrants”/

8. Internationality/)

9. (resettlement or re-settlement or border crossing* or newcomer* or naturali?ed citizen or permanent residen* or nonnative* or non native* or settler* or new arrival* or displaced person* or displaced people* or foreigner* or foreign born or foreign adopt* or migration or migrant* or immigrant* or immigration or emigrant* or emigration or international* adopt* or intercountr* adopt* or inter countr* adopt*).tw.

10. exp Refugees/

11. (asylum seeker* or refugee* or alien*).tw.

12. or/6-12 [Immigrant population terms]

13. 5 and 12

14. Limit 13 to Animals/ not Humans/

15. 13 not 14

16. Remove duplicates from 15

Note:

The above strategy is designed for the OvidSP platform for MEDLINE.

Terms ending with a forward slash (/) are MeSH (Medical Subject Headings).

*exp* indicates that a MeSH has been exploded to include narrower search terms in the subject heading hierarchy

The asterisk (*) indicates truncation, where the stem of the word is searched with or without additional suffixes.

The question mark (?) indicates the presence of any letter (e.g., z or s).

The command *.tw.* indicates that term is searched as a textword, within the title or abstract fields.
