# Supplementary material for: The Seroprevalence of Hepatitis C Antibodies in Immigrants and Refugees from Intermediate and High Endemic Countries: A Systematic Review and Meta-Analysis
Source: PLoS One. 2015 Nov 11;10(11):e0141715. doi: 10.1371/journal.pone.0141715 (PMC4641717; doi:10.1371/journal.pone.0141715)
Supplement: S3 Appendix — (DOCX) [file pone.0141715.s003.docx]

**Supporting Information 3**

**Quality Assessment of Included Studies**

|  |  | **Quality Assessment** | | | | |  | |
| --- | --- | --- | --- | --- | --- | --- | --- | --- |
| **Outcome** | **Study Design** | **Number of Studies** | **Risk of Bias** | **Inconsistency** | **Indirectness** | **Imprecision** | **OVERALL QUALITY** | **IMPORTANCE** |
| Principle outcome:  HCV Prevalence | Observational | 50 | Selection Low/moderate | Moderate to high | Low | Low to moderate | Low to Moderate | Important |
|  | Observational | 20 | Detection bias  Low |  |  |  |  |  |
|  | Observational | 30 | Detection bias  Moderate |  |  |  |  |  |
| HCV PCR | Observational | 6 | Moderate | High | Low | High | Very Low | Very important |

**Selection Bias:**

*Low to moderate:* all subjects or a random sample of subjects of non-population based samples captured in a particular setting but usually in a single primary care or immigrant clinic, refugee reception center, pre-natal clinic etc. or ≥ 60% participation rate in a survey.

*High:* Non-random exclusion of subjects in a particular setting or <60% participation in a survey. Migrants at lower (blood donors)

or higher risk for HCV (sex workers, intravenous drug use, incarceration, individuals being treated for chronic liver disease etc.)

**Detection Bias:**

*Low:* All subjects tested and all enzyme immunoassays confirmed with a RIBA/Immunoblot or PCR.

*Moderate:* All subjects tested but method of antibody testing not mentioned or not confirmed

*High:* Only a subset of subjects tested.

**Inconsistency:** Moderate to high as the heterogeneity of the overall pooled estimate was high but decreased with stratification by age group and region of origin, know important predictors of HCV prevalence

**Indirectness:** Low due to inclusion of only studies of migrant populations that were felt to be representative of the general migrant population

**Imprecision:** Low to moderate given relatively narrow confidence intervals for the overall estimate but increased in stratified analyses by age and region of origin

**Publication bias:** Not applicable to meta-analysis of prevalence studies
