# Supplementary material for: The Seroprevalence of Hepatitis C Antibodies in Immigrants and Refugees from Intermediate and High Endemic Countries: A Systematic Review and Meta-Analysis
Source: PLoS One. 2015 Nov 11;10(11):e0141715. doi: 10.1371/journal.pone.0141715 (PMC4641717; doi:10.1371/journal.pone.0141715)
Supplement: S4 Appendix — (DOCX) [file pone.0141715.s004.docx]

# S4. Estimated HCV Seroprevalence (previous and active) in Host country and Region of Origin

**Table A- List of the 24 included high income migrant-receiving countries with low/intermediate HCV seroprevalence from published literature**

| **Country** | **HCV Seroprevalence, %** | | |
| --- | --- | --- | --- |
|  | **WHO**[**^1^**](#_ENREF_1) | **ECDC**[**^2^**](#_ENREF_2) | **Cornberg et al.**[**^3^**](#_ENREF_3) |
| **North America** |  |  |  |
| Canada | 1.0 | - | 1.0 |
| United States | 1.8 | - | - |
| **Europe** |  |  |  |
| Austria | 1.0 | - | - |
| Belgium | 0.9 | 0.6 |  |
| Czech Republic | 1.5 | - | 1.5-2.0 |
| Denmark | 0.5 | - | - |
| Finland | 0.5 | - | - |
| France | 1.3 | 0.8 | 1.3 |
| Germany | 0.8 | 0.4 | 0.4 |
| Greece | 1.5 | 1.0 | 1.0-1.9 |
| Iceland | 0.5 | - | - |
| Republic of Ireland | 1.1 | - | - |
| Israel | 1.0 | - | 2.0 |
| Italy | 3.2 | 5.2 | 5.2 |
| Luxembourg | 1.0 | - | - |
| Netherlands | 1.0 | 0.4 | - |
| Norway | 0.6 | - | 0.7 |
| Portugal | 1.0 | - | 1.5 |
| Spain | 2.0 | 2.0 | 2.6 |
| Sweden | 0.5 | 0.4 | 0.6 |
| Switzerland | 1.0 | - | 1.3-1.8 |
| United Kingdom | 1.1 | 0.7 | 0.6-1.0 |
| **Oceania** |  |  |  |
| Australia | 1.1 | - | - |
| New Zealand | 0.3 | - | - |

**Table B** **– HCV seroprevalence estimates from the World Health Organization**[**^1^**](#_ENREF_1) **(unless specified otherwise) for countries of origin by World Bank region.**

| **East Asia and Pacific (Weighted seroprevalence = 2.32) ^*^** | | | |
| --- | --- | --- | --- |
|  | | | |
| **Country** | **HCV (%)**† | **Country** | **HCV (%)** |
| American Samoa | 2.0 | Northern Mariana Islands | - |
| Cambodia | 4.1 | Pacific Islands | - |
| China | 2.2; 1-1.9[^4^](#_ENREF_4) | Palau | 2.0 |
| Fiji | 2.0 | Papua New Guinea | 2.0 |
| Indonesia | 3.9 | Philippines | 2.2 |
| Japan | 2.4; 1-1.9[^4^](#_ENREF_4) | Samoa | 0.8 |
| Kiribati | 2.0 | Singapore | 1.0 |
| Korea, Dem. Rep. | 1.0; 1.3[^4^](#_ENREF_4) | Solomon Islands | 2.0 |
| Lao PDR | 1.1 | Taiwan | 4.4[^4^](#_ENREF_4) |
| Malaysia | 1.5 | Thailand | 2.2; 2.8[^4^](#_ENREF_4) |
| Marshall Islands | 1.5 | Timor-Leste | - |
| Micronesia, Fe. Sts | 2.0 | Tonga | 2.0 |
| Mongolia | 10.7 | Vanuatu | 2.0 |
| Myanmar | 1.0 | Vietnam | 1.0; 2-2.9[^4^](#_ENREF_4) |

* Weighted averages were calculated using the WHO estimated HCV seroprevalence and the 2009 national population estimates from the World Bank[^5^](#_ENREF_5)

† HCV seroprevalence estimates represent antibody positivity

| **Eastern Europe and Central Asia (Weighted seroprevalence = 3.46)** | | | |
| --- | --- | --- | --- |
|  | | | |
| **Country** | **HCV (%)** | **Country** | **HCV (%)** |
| Albania | 1.5 | Lithuania | 2.2 |
| Armenia | 4.0 | Macedonia, FYR | 2.0 |
| Azerbaijan | 4.0 | Moldova | 2.3 |
| Belarus | 2.2 | Montenegro | 1.5 |
| Bosnia and Herzegovina | 1.5 | Poland | 2.0; 1.9[^2^](#_ENREF_2)^,^[^3^](#_ENREF_3) |
| Bulgaria | 1.8; 1.3[^2^](#_ENREF_2) | Romania | 4.5; 3.5[^2^](#_ENREF_2)^,^[^3^](#_ENREF_3) |
| Croatia | 1.5 | Russian Federation | 4.1; 2.0-2.5[^3^](#_ENREF_3) |
| Estonia | 5.0 | Serbia | 1.5 |
| Georgia | 6.7 | Slovak Republic | 1.0 |
| Hungary | 2.2; 0.6[^3^](#_ENREF_3) | Tajikistan | 4.0 |
| Kazakhstan | 3.2 | Turkey | 2.2; 1.0-1.5[^3^](#_ENREF_3) |
| Kosovo | - | Turkmenistan | 4.0 |
| Kyrgyz Republic | 4.0 | Ukraine | 4.0 |
| Latvia | 2.2 | Uzbekistan | 6.5 |

| **Latin America and the Caribbean (Weighted seroprevalence = 1.32)** | | | |
| --- | --- | --- | --- |
|  | | | |
| **Country** | **HCV (%)** | **Country** | **HCV (%)** |
| Antigua and Barbuda | 0.8 | Guyana | 0.8 |
| Argentina | 1.9; 1.5[^6^](#_ENREF_6) | Haiti | 4.4 |
| Belize | 0..8 | Honduras | 0.5 |
| Bolivia | 4.7 | Jamaica | 0.8 |
| Brazil | 1.4; 1.5[^6^](#_ENREF_6) | Mexico | 1.0; 1.0[^6^](#_ENREF_6) |
| Chile | 0.9 | Nicaragua | 0.4 |
| Colombia | 1.0 | Panama | 0.8 |
| Costa Rica | 0.8 | Paraguay | 1.2 |
| Cuba | 1.8 | Peru | 1.0 |
| Dominica | 0.8 | St. Kitts and Nevis | 2.2 |
| Dominican Republic | 0.8 | St. Lucia | 0.8 |
| Ecuador | 1.4 | St. Vincent and the Grenadines | 1.0 |
| El Salvador | 2.5 | Suriname | 0.8 |
| Grenada | 5.0 | Uruguay | 1.0 |
| Guatemala | 0.8 | Venezuela, RB | 0.9 |
| **Middle East and North Africa (Weighted seroprevalence = 4.20/1.33^a^)** | | | |
|  | | | |
| **Country** | **HCV (%)** | **Country** | **HCV (%)** |
| Algeria | 0.2 | Morocco | 1.9 |
| Bahrain | 1.8 | Oman | 1.2 |
| Djibouti | 0.3 | Qatar | 1.8 |
| Egypt, Arab Rep. | 14.0; 14.9[^4^](#_ENREF_4) | Syrian Arab Republic | 0.5 |
| Iran, Islamic Rep. | 0.9 | Tunisia | 1.2 |
| Iraq | 3.2 | United Arab Emirates | 2.3 |
| Jordan | 2.1 | West Bank and Gaza | - |
| Lebanon | 0.7 | Yemen, Rep. | 1.7 |
| Libya | 1.6 |  |  |

^a^Excludes Egypt

| **South Asia (Weighted seroprevalence = 1.85)** | | | |
| --- | --- | --- | --- |
|  | | | |
| **Country** | **HCV (%)** | **Country** | **HCV (%)** |
| Afghanistan | 0.5 | Maldives | 1.0 |
| Bangladesh | 0.6 | Nepal | 0.6 |
| Bhutan | 1.3 | Pakistan | 5.9; 4.7[^4^](#_ENREF_4) |
| India | 1.5; 1-1.9[^4^](#_ENREF_4) | Sri Lanka | 1.0 |

| **Sub-Saharan Africa (Weighted seroprevalence = 3.28)** | | | |
| --- | --- | --- | --- |
|  | | | |
| **Country** | **HCV (%)** | **Country** | **HCV (%)** |
| Angola | 5.0 | Madagascar | 1.7; 2.1[^7^](#_ENREF_7) |
| Benin | 1.6 | Malawi | 6.8; 0.7[^7^](#_ENREF_7) |
| Botswana | 1.6 | Mali | 3.3 |
| Burkina Faso | 5.2; 4.9[^7^](#_ENREF_7) | Mauritania | 1.1 |
| Burundi | 11.3 | Mauritius | 2.1 |
| Cameroon | 13.8 | Mayotte | - |
| Cape Verde | 3.0 | Mozambique | 3.2; 2.8[^7^](#_ENREF_7) |
| Central African Republic | 2.4 | Namibia | 0.9 |
| Chad | 5.0; 4.8[^7^](#_ENREF_7) | Niger | 3.2; 1.8[^7^](#_ENREF_7) |
| Comoros | 1.0 | Nigeria | 2.1 |
| Congo, De. Rep. | 6.4; 5.5[^7^](#_ENREF_7) | Rwanda | 4.9; 4.1[^7^](#_ENREF_7) |
| Congo, Rep. | - | São Tomé and Principe | 10.0 |
| Côte d’Ivoire | 3.3 | Senegal | 3.0; 2.2[^7^](#_ENREF_7) |
| Equatorial Guinea | 1.7 | Seychelles | 0.3 |
| Eritrea | 1.9 | Sierra Leone | 2.0 |
| Ethiopia | 1.9 | Somalia | 1.0; 1.5[^7^](#_ENREF_7) |
| Gabon | 9.2 | South Africa | 1.7; 0.1[^7^](#_ENREF_7) |
| Gambia, The | 2.4 | Sudan | 2.8 |
| Ghana | 1.7 | Swaziland | 1.5 |
| Guinea | 5.5 | Tanzania | 3.2 |
| Guinea-Bissau | 4.7 | Togo | 3.3; 3.9[^7^](#_ENREF_7) |
| Kenya | 0.9 | Uganda | 6.6 |
| Lesotho | 1.0 | Zambia | 0.2 |
| Liberia | 3.0 | Zimbabwe | 2.0 |

**References**

1. World Health Organization. Hepatitis C - global prevalence (Update). *Wkly Epidemiol Rec.* Dec 10 1999;74(49):425-427.

2. European Centre for Disease Prevention and Control. *Hepatitis B and C in the EU neighbourhood: prevalence, burden of disease and screening policies.* Stockholm: ECDC;2010.

3. Cornberg M, Razavi HA, Alberti A, et al. A systematic review of hepatitis C virus epidemiology in Europe, Canada and Israel. *Liver Int.* 2011;31 Suppl 2:30-60.

4. Sievert W, Altraif I, Razavi HA, et al. A systematic review of hepatitis C virus epidemiology in Asia, Australia and Egypt. *Liver Int.* July 2011;31(SUPPL. 2):61-80.

5. The World Bank. Countries and Economies. 2014; <http://data.worldbank.org/country>. Accessed January 13, 2014.

6. Kershenobich D, Razavi H, A., Sanchez-Avila J, F., et al. Trends and projections of hepatitis C virus epidemiology in Latin America. *Liver Int.* Jul 2011;31(Suppl 2):18-29. doi: 10.1111/j.1478-3231.2011.02538.x.

7. Madhava V, Burgess C, Drucker E. Epidemiology of chronic hepatitis C virus infection in sub-Saharan Africa. *Lancet Infect Dis.* 2002;2(5):293-302.
