# Supplementary figures and images for: The Seroprevalence of Hepatitis C Antibodies in Immigrants and Refugees from Intermediate and High Endemic Countries: A Systematic Review and Meta-Analysis
Source: PLoS One. 2015 Nov 11;10(11):e0141715. doi: 10.1371/journal.pone.0141715 (PMC4641717; doi:10.1371/journal.pone.0141715)

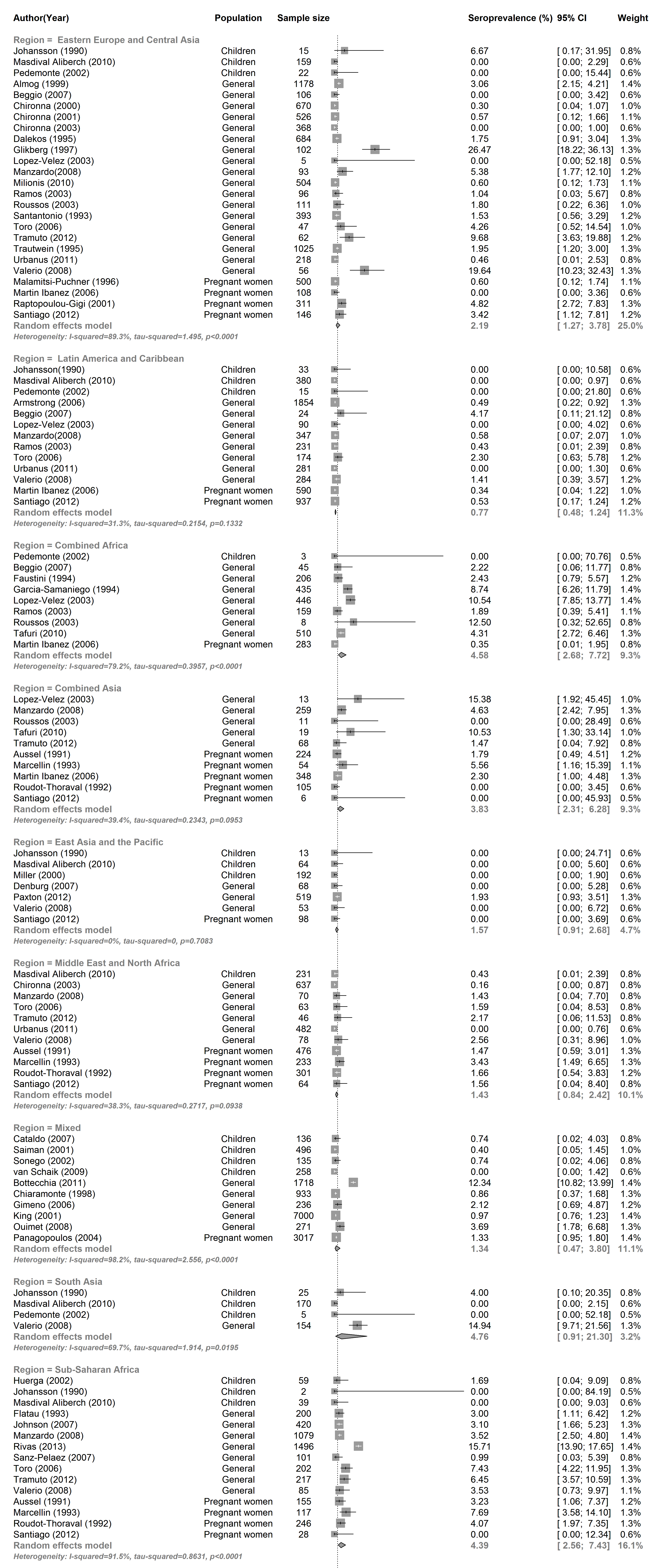

Supplement: S1 Fig — (TIFF) [file pone.0141715.s005.tiff]
