# Supplementary figures and images for: Correction: The Seroprevalence of Hepatitis C Antibodies in Immigrants and Refugees from Intermediate and High Endemic Countries: A Systematic Review and Meta-Analysis
Source: PLoS One. 2015 Dec 9;10(12):e0144567. doi: 10.1371/journal.pone.0144567 (PMC4674089; doi:10.1371/journal.pone.0144567)

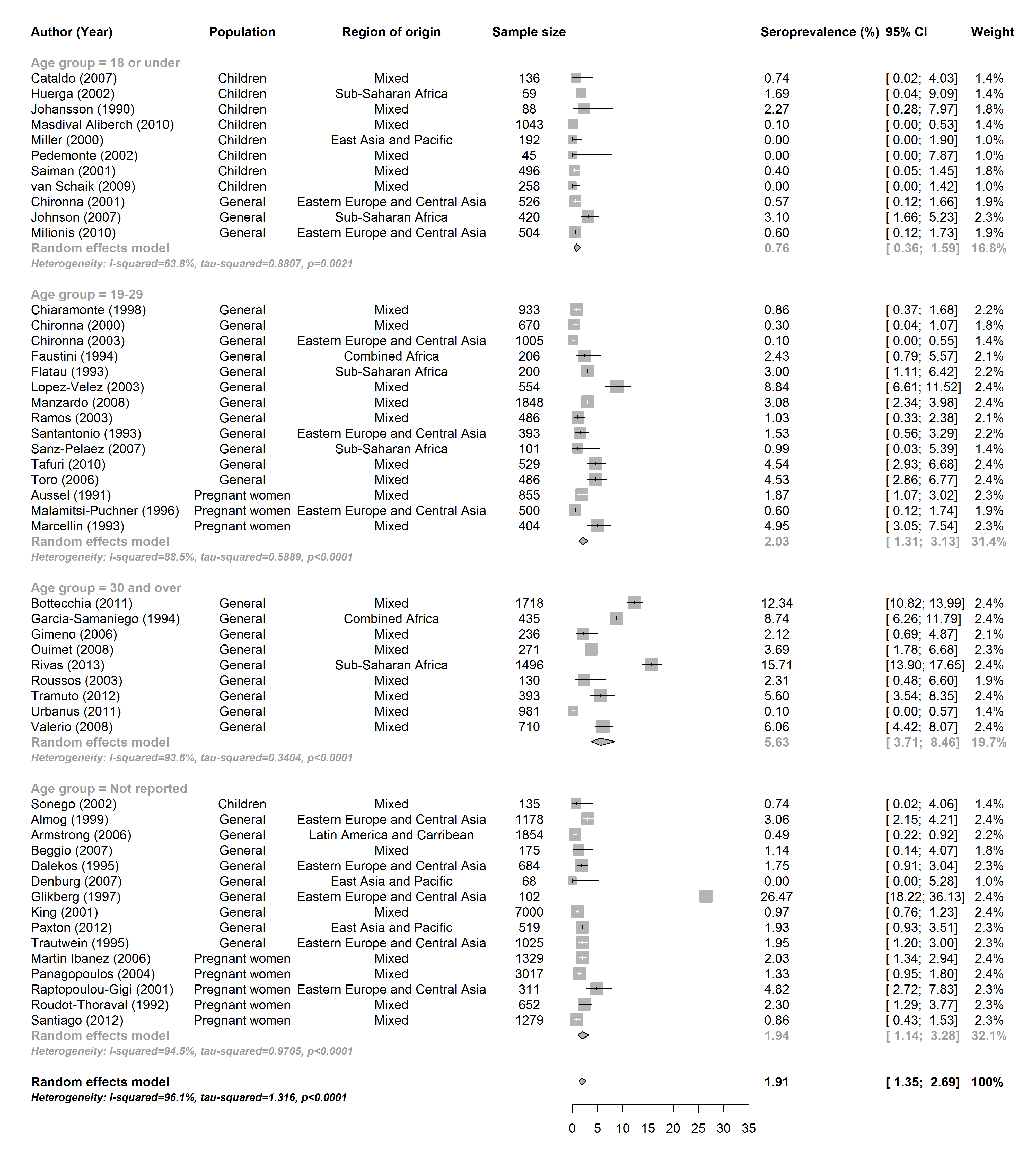

Supplement: S1 Fig — This file includes supplementary data. (TIFF) [file pone.0144567.s001.tiff]
